# Pkd2l1 deletion inhibits the neurogenesis of cerebrospinal fluid-contacting neurons and impedes spinal cord injury repair

**DOI:** 10.1038/s41420-025-02492-y

**Published:** 2025-04-23

**Authors:** Yi Zhang, Liang Cao, Haijian Yan, Zhangrong Luo, Chanjuan Chen, Zeyu Shangguan, Qizhe Li, Xuexing Shi, Leiluo Yang, Wei Tan, Shengxin Yang, Jiangquan Fu, Chunqing Wang, Xiaowei Dou, Qing Li

**Affiliations:** 1 Emergency Department, Emergency Medicine Laboratory, The Affiliated Hospital of Guizhou Medical University, Guiyang, China; 2 School of Clinical Medicine, Guizhou Medical University, Guiyang, China; 3 Shunyi Maternal and Children’s Hospital of Beijing Children’s Hospital, Beijing, China; 4 Clinical Research Center, Affiliated Hospital of Guizhou Medical University, Guiyang, China

**Keywords:** Adult neurogenesis, Neural stem cells

## Abstract

Adult neural stem cells (NSCs) offer a promising avenue for restoring spinal cord injury (SCI). However, their precise identity in the mammalian spinal cord remains unclear. Our previous research demonstrated that Pkd2l1-positive cerebrospinal fluid-contacting neurons (CSF-cNs) possess the NSC properties. Furthermore, understanding the role and molecular mechanisms of CSF-cNs as endogenous NSCs in spinal cord repair is crucial for developing effective treatments. This study utilizes a Pkd2l1^−/−^ transgenic mouse model to investigate the role of CSF-cNs in SCI repair. We found that the CSF-cN population was almost absent in Pkd2l1^−/−^ mice. Following SCI, these mice exhibited a significant reduction in the number of NSCs surrounding the central canal. Notably, Pkd2l1^−/−^ mice showed impaired neuronal regeneration and compromised motor function recovery post-SCI. These findings highlight the potential importance of Pkd2l1 as a target for treating SCI by focusing on endogenous NSCs.

## Introduction

Adult neural stem cells (NSCs) in the adult mammalian spinal cord provide exciting prospects for spinal cord injury (SCI) repair, but their actual identity has remained elusive [1–5]. Adult NSCs reside in the subventricular zone of the spinal cord, which acts as the neurogenic niche [6–9]. The spinal neurogenic niche is now supposed to primarily compose of ependymocytes and cerebrospinal fluid–contacting neurons (CSF-cNs). In response to SCI, proliferation of ependymal cells increases dramatically, and these cells can differentiate into astrocytes, suggesting that ependymal cells are NSCs [10–12]. However, the acquisition of neurogenic function by ependymal cells remains controversial [4]. Specifically, using a transgenic mouse model with targeted labeling, ependymal cells were confirmed to lack the ability to form neurospheres in vitro, and ventricular injection of basic fibroblast growth factor (bFGF) and vascular endothelial growth factor (VEGF) failed to activate adult ependymal cell as NSCs, implying that ependymal cells do not have latent NSC function [13]. Therefore, these findings imply that whether spinal cord ependymal cells possess neural stem cell properties remains unresolved. In contrast, our study demonstrated that mouse Pkd2l1-positive CSF-cNs form neurospheres and can differentiate into neurons, astrocytes, and oligodendrocytes in vitro [14]. SCI or injection of the neurotrophic factors VEGF and bFGF into the lateral ventricle promotes the proliferation of CSF-cNs in vivo [15]. These results suggest that CSF-cNs act as NSCs in the adult spinal cord [16]. Moreover, recent research has revealed that CSF-cNs are the lone subpopulation to exhibit statistically significant resilience following SCI [17]. In conclusion, increasing evidence suggests that CSF-cNs may be a valuable target for endogenous spinal cord repair [16]. In-depth research on the role of CSF-cNs as endogenous NSCs in spinal cord repair, along with their molecular mechanisms, is crucial for their application in the treatment of SCI.

Polycystic kidney disease 2 like 1 (Pkd2l1), a specific marker of CSF-cNs, plays a fundamental role in sensing various environmental and cellular signals, including chemical changes and pressure [18–22]. Pkd2l1 was first found to be expressed in mammalian taste cells as a sour taste sensor and in CSF-cNs around the central canal of the spinal cord as an extracellular pH sensor [18]. Recent evidence indicates that Pkd2l1 functions as a mechanosensory in CSF-cNs, and Pkd2l1 mutation results in exaggerated spine curvature in adult zebrafish [20]. In addition, Pkd2l1 functions as a Ca^2+^ channel, and the intracellular Ca^2+^ concentration can activate NSCs [23–25]. However, the exact role of Pkd2l1 in regulating CSF-cN and in spinal cord repair is still unknown.

In this study, we explored the role of Pkd2l1 in regulating the CSF-cN population and SCI repair in mice. We found that Pkd2l1 deletion resulted in almost complete depletion of the CSF-cN population. Interestingly, the ablation of CSF-cNs through Pkd2l1 deletion suppressed cell proliferation and NSCs surrounding the central canal of spinal cord, indicating that CSF-cNs may be the main source of NSCs after SCI. Consistent with this finding, Pkd2l1^−/−^ mice showed impaired neurogenesis and deficient motor recovery after SCI. Our results demonstrate that Pkd2l1 is a vital regulator of CSF-cNs neurogenesis and plays a key role in repair after SCI.

## Results

### CSF-cNs were confirmed as NSCs

**Fig. 1:**
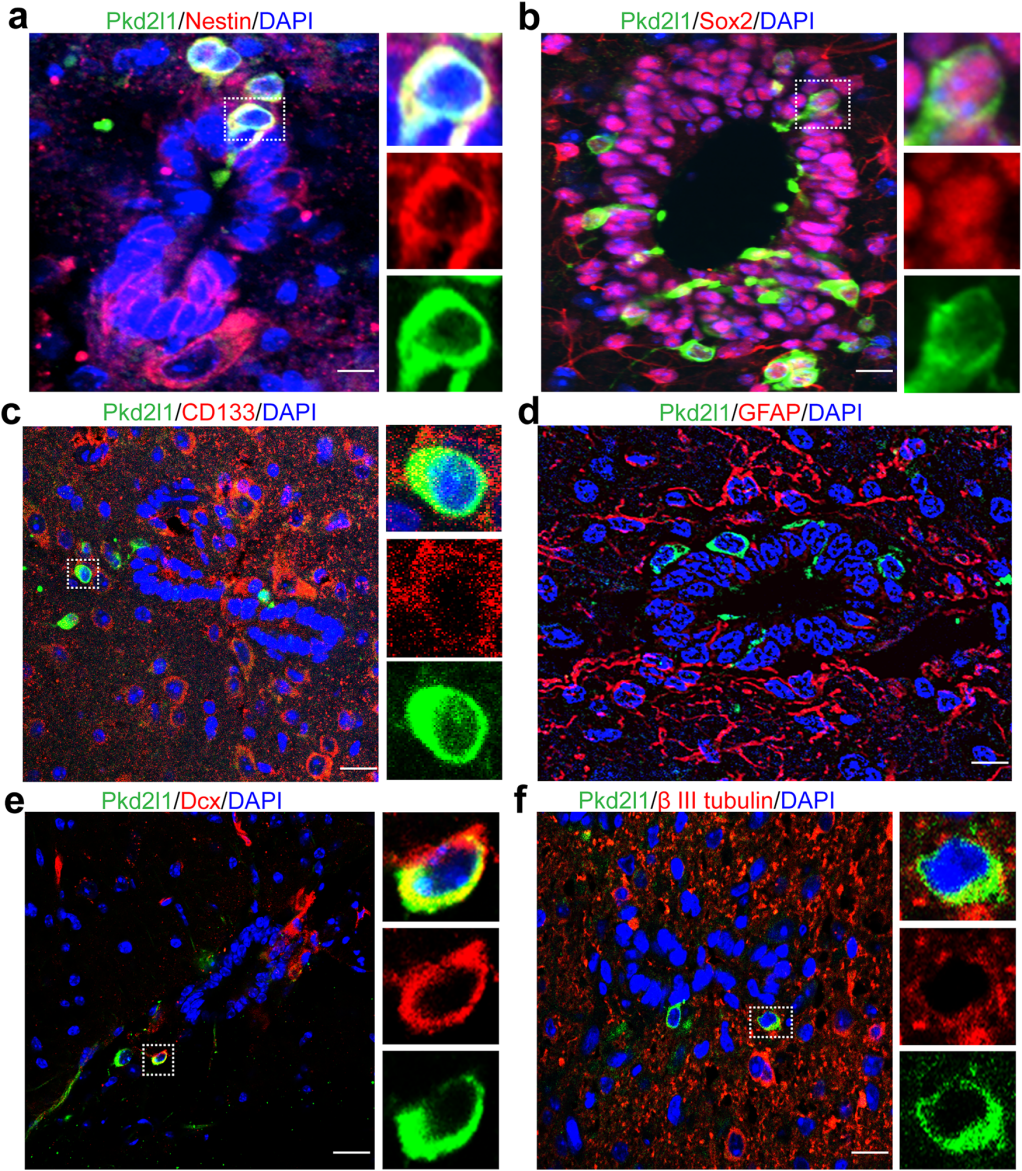
Pkd2l1^+^ CSF-cNs have the characteristics of both immature neurons and adult NSCs. **a** Immunofluorescence staining showing expression of the NSC marker Nestin in Pkd2l1^+^ CSF-cNs. **b** Pkd2l1^+^ CSF-cNs expressed the NSC marker Sox2 by immunofluorescence staining. **c** Pkd2l1^+^ CSF-cNs expressed the NSC marker CD133 by immunofluorescence staining. **d** Pkd2l1^+^ CSF-cNs did not express the NSC marker GFAP by immunofluorescence staining. **e** Pkd2l1^+^ CSF-cNs expressed the neuroblast marker doublecortin (Dcx) by immunofluorescence staining. **f** Immunofluorescence staining showed that Pkd2l1^+^ CSF-cNs expressed the immature neuronal marker βIII-tubulin (red). Scale bars, 20 µm. Nuclear counterstaining, DAPI.

CSF-cNs express high levels of immature neuron markers such as Sox2, doublecortin (Dcx) and β-III tubulin [26, 27]. We also previously confirmed that CSF-cNs have properties of NSCs in vitro and in vivo [14, 15]. To confirm that CSF-cNs act as NSCs, we assessed the expression of NSC markers in CSF-cNs of mice spinal cord sections by immunofluorescence assay. CSF-cNs expressed the typical NSC markers Nestin, Sox2, and CD133 (Fig. 1a–c), but did not express the NSC marker GFAP (Fig. 1d). In addition, CSF-cNs expressed the immature neurons markers Dcx and β-III tubulin (Fig. 1e, f). These results suggest that CSF-cNs are NSCs.

### Pkd2l1 deletion suppresses CSF-cNs neurogenesis

**Fig. 2:**
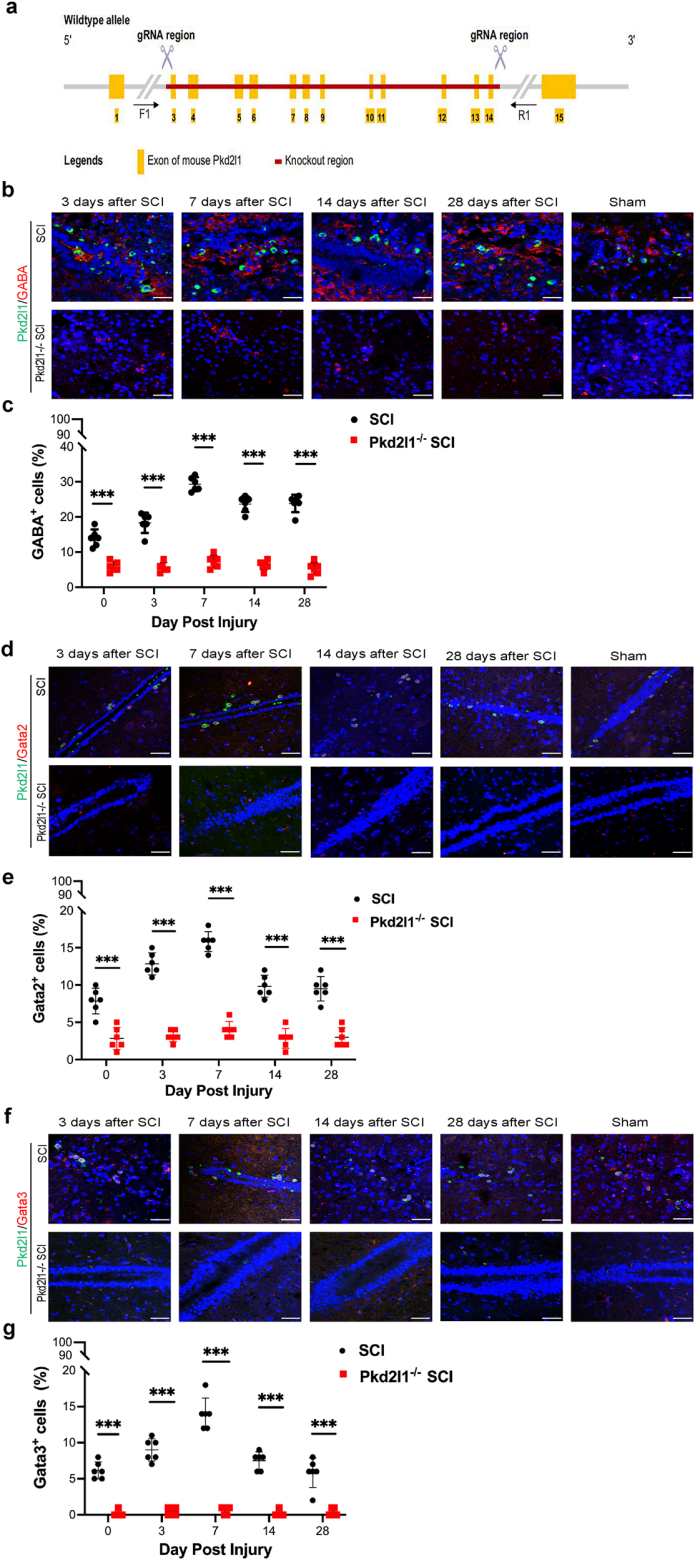
Pkd2l1 deletion results in the loss of CSF-cNs. **a** Schematic showing the generation and identification of genetically homogeneous Pkd2l1^−/−^ mice. **b**, **c** Immunofluorescence staining of spinal cord sections showed that Pkd2l1 deletion caused the obviously reduced proliferation of GABA-positive cells after SCI (GABA: CSF-cNs marker) (*n* = 6). **d**, **e** The depletion of Pkd2l1 caused a significant reduction in Gata2-positive cells after SCI, as shown by immunofluorescence staining (Gata2: CSF-cNs marker) (*n* = 6). **f**, **g** Immunofluorescence staining shows that Pkd2l1 deletion resulted in almost the complete absence of Gata3-positive cell proliferation after SCI (Gata3: CSF-cNs marker) (*n* = 6). ***p < 0.001. Data are presented as the mean ± SD. Scale bars, 50 µm. Nuclear counterstaining, DAPI.

Since Pkd2l1 functions as a Ca^2+^ ion channel and intracellular Ca^2+^ elevation promotes the proliferation of NSCs [24, 28], we explored the effect of Pkd2l1 deletion on CSF-cNs neurogenesis using Pkd2l1^−/−^ mice (Fig. 2a). Because CSF-cNs are GABAergic neurons in the ventral part of the spinal cord and GABA exhibits potential as a marker for CSF-cNs [19, 29, 30], we assessed CSF-cNs neurogenesis by GABA expression on CSF-cNs in Pkd2l1^−/−^ mice through immunofluorescence staining. Immunofluorescence analysis of GABA expression showed that the population of GABAergic neurons was significantly reduced and the proliferation of GABAergic neurons was evidently inhibited in Pkd2l1^−/−^ mice compared with wild-type (WT) mice after SCI (*p* < 0.001, *n* = 6, Fig. 2b, c), indicating that Pkd2l1 deletion decreases the population of CSF-cNs. To further verify regulation of the CSF-cN population by Pkd2l1, we also used immunofluorescence to evaluate Gata2 and Gata3, which are potential markers of CSF-cNs [27]. Similar to the results obtained for GABA, Pkd2l1^−/−^ mice displayed a significant decrease in the population of Gata2/3^+^ cells (*p* < 0.001, *n* = 6, Fig. 2d, e) and suppressed Gata2/3^+^ cell population (*p* < 0.001, *n* = 6, Fig. 2f, g), suggesting that the depletion of Pkd2l1 suppresses CSF-cN reduces the CSF-cN population. Together, these results suggest that Pkd2l1 deletion results in the near complete depletion of CSF-cNs and that Pkd2l1 may be a vital regulator of CSF-cN neurogenesis.

### Pkd2l1 deletion restrains the proliferation and migration of CSF-cNs

**Fig. 3:**
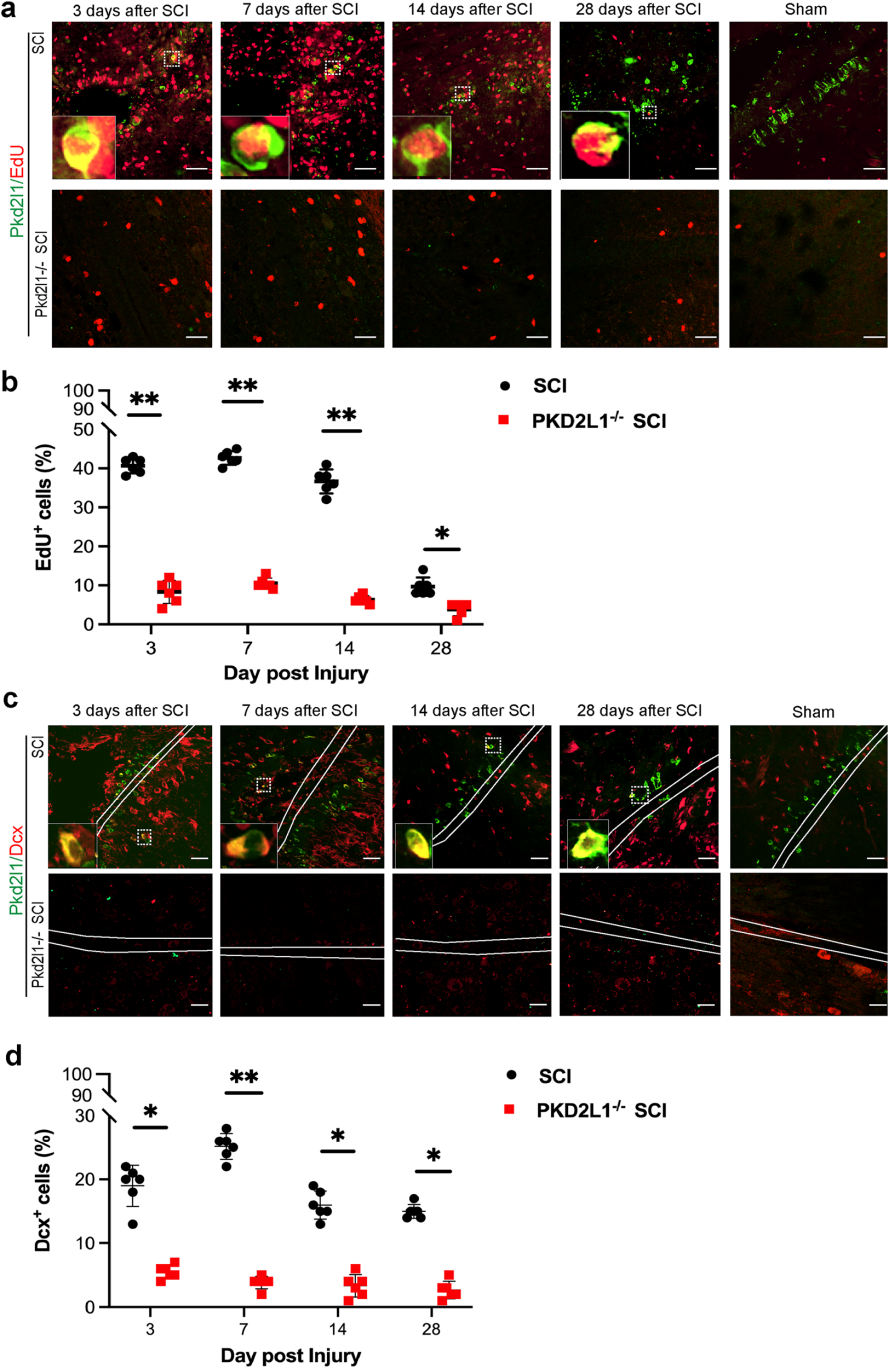
Pkd2l1 deletion suppresses the proliferation of CSF-cNs and leads to the loss of migratory CSF-cNs following SCI. **a**, **b** The EdU proliferation assay revealed a significant reduction in EdU-positive cells in Pkd2l1^−/−^ mice compared to wild-type counterparts (*n* = 6). **c**, **d** Immunofluorescence analysis revealed a notable decrease in Dcx-positive cells following SCI in Pkd2l1^−/−^ mice compared to their wild-type counterparts (*n* = 6). *p < 0.05, **p < 0.01. Data are presented as the mean ± SD. Scale bars, 50 µm.

After confirming that Pkd2l1 regulates CSF-cN neurogenesis, we sought to next clarify the role of Pkd2l1 in CSF-cN proliferation. To determine the role of Pkd2l1 in regulating CSF-cN proliferation, an EdU proliferation assay was performed in tissue slices of CSF-cNs from Pkd2l1^−/−^ transgenic mice after SCI. The results showed that after SCI, the number of EdU^+^ cells surrounding the central canal of the spinal cord in WT mice significantly increased (*p* < 0.01, *n* = 6, Fig. 3a, b). However, it remains uncertain from this experiment whether these EdU^+^ cells are neural cells or belong to other cell types. Notably, the number of EdU^+^/Pkd2l1^+^ cells in WT mice increased compared to the sham group (Fig. 3a). These findings suggest that Pkd2l1 plays a critical role in the proliferation of cells around the central canal after SCI, while also implying that CSF-cN may serve as the primary proliferative cell population in this region following SCI.

To unravel the role of Pkd2l1 in CSF-cN migration, immunofluorescence staining was performed to detect the expression of Dcx, a microtubule-associated protein specifically expressed in migrating neurons during embryonic and postnatal development of the central and peripheral nervous systems [31], in WT and Pkd2l1^−/−^ mice after SCI. We found that the proportion of migratory Dcx^+^ cell clearly increased in WT mice after SCI, particularly increasing by 3 days to a maximum at 7 days after SCI (*p* < 0.05, *n* = 6, Fig. 3c, d). Nevertheless, Pkd2l1 deletion obviously reduced the proportion of migratory neural cells surrounding the central canal following SCI (Fig. 3c). These data indicate that Pkd2l1 plays a central role in CSF-cN migration surrounding the central canal following SCI.

### Ablation of CSF-cNs by Pkd2l1 deletion suppresses NSC proliferation surrounding the central canal after SCI

**Fig. 4:**
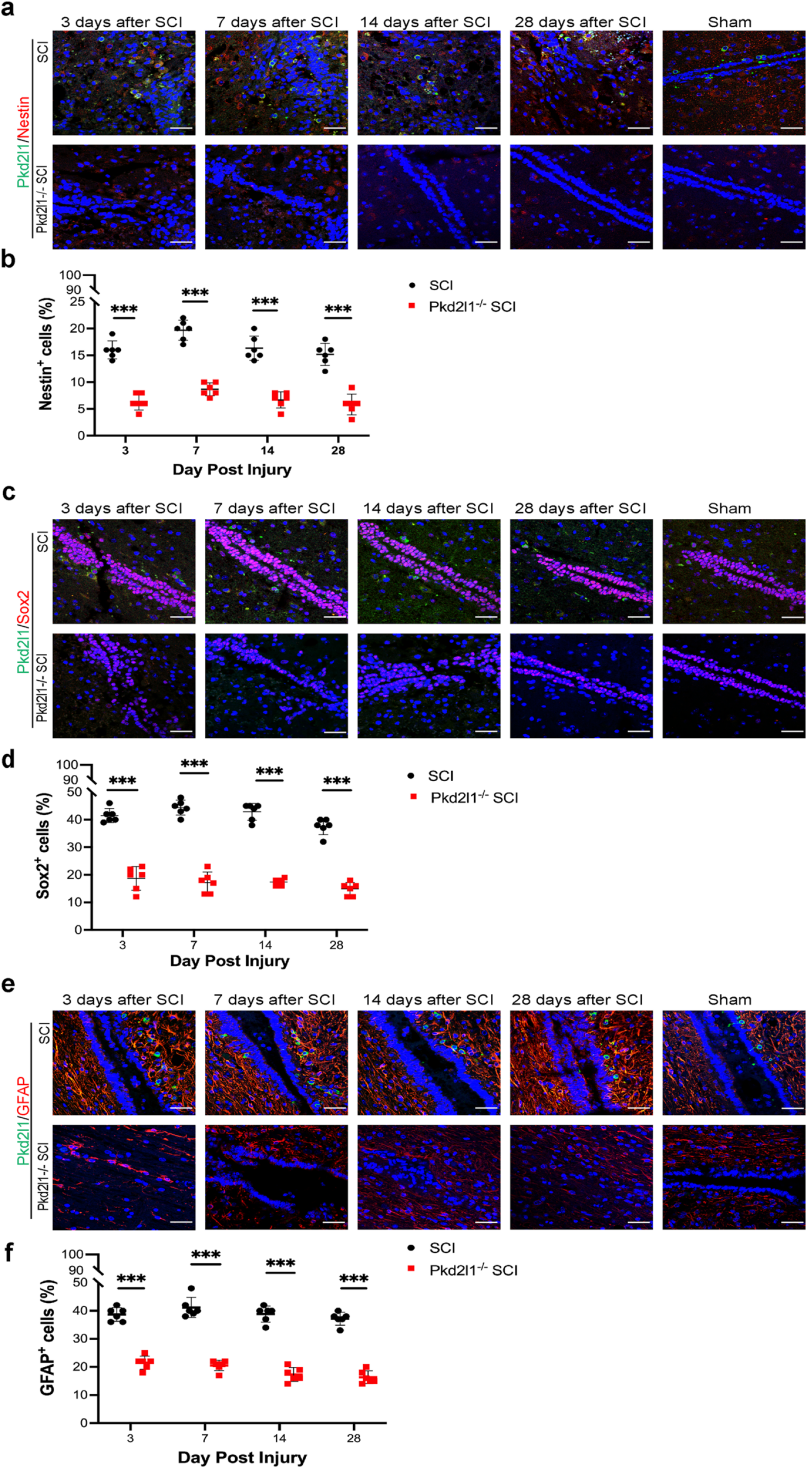
Pkd2l1 deletion retards the self-renewal of NSCs surrounding the central canal of the spinal cord after SCI. **a**, **b** Immunofluorescence staining showed that Pkd2l1^−/−^ mice had almost no Nestin-positive cells surrounding the central canal of the spinal cord after SCI (*n* = 6). **c**, **d** Sox2 expression evidently decreased in Pkd2l1^−/−^ mice after SCI, as shown by immunofluorescence staining (*n* = 6). **e**, **f** GFAP expression was reduced in Pkd2l1^−/−^ mice after SCI, as shown by immunofluorescence staining (*n* = 6). ***p < 0.001. Data are presented as the mean ± SD. Scale bars, 50 µm. Nuclear counterstaining, DAPI.

Since Pkd2l1 mutation in CSF-cNs causes Ca^2+^ channel paralysis and Ca^2+^ channels are involved in the proliferation of NSCs [32], we inferred that Pkd2l1 deletion in CSF-cNs inhibits the proliferation of NSCs surrounding the central canal. To determine whether Pkd2l1 deletion in CSF-cNs affect the proliferation of NSCs surrounding the central canal following SCI, immunofluorescence assay was done in Pkd2l1^−/−^ mice and WT mice. Immunofluorescence staining results revealed that the expression of Nestin, an NSC marker, surrounding the central canal increased after SCI and was particularly higher at 7 days after SCI in WT mice (*p* < 0.001, *n* = 6, Fig. 4a, b). Comparatively, in Pkd2l1^−/−^ transgenic mice, Pkd2l1 deletion suppressed Nestin expression surrounding the central canal after SCI (*p* < 0.001, *n* = 6, Fig. 4a, b). These results suggest that the loss of Pkd2l1 in CSF-cNs suppresses the proliferation of NSCs surrounding the central canal after SCI. Similar results were obtained for the other NSC markers Sox2 and GFAP (*p* < 0.001, *n* = 6, Fig. 4c–f). Taken together, our results show that Pkd2l1 deletion in CSF-cNs leads to a decrease in NSC proliferation after SCI and indicates that CSF-cNs may be the primary NSCs surrounding the central canal of the spinal cord.

### Pkd2l1 deletion inhibits neural regeneration and repair following SCI

**Fig. 5:**
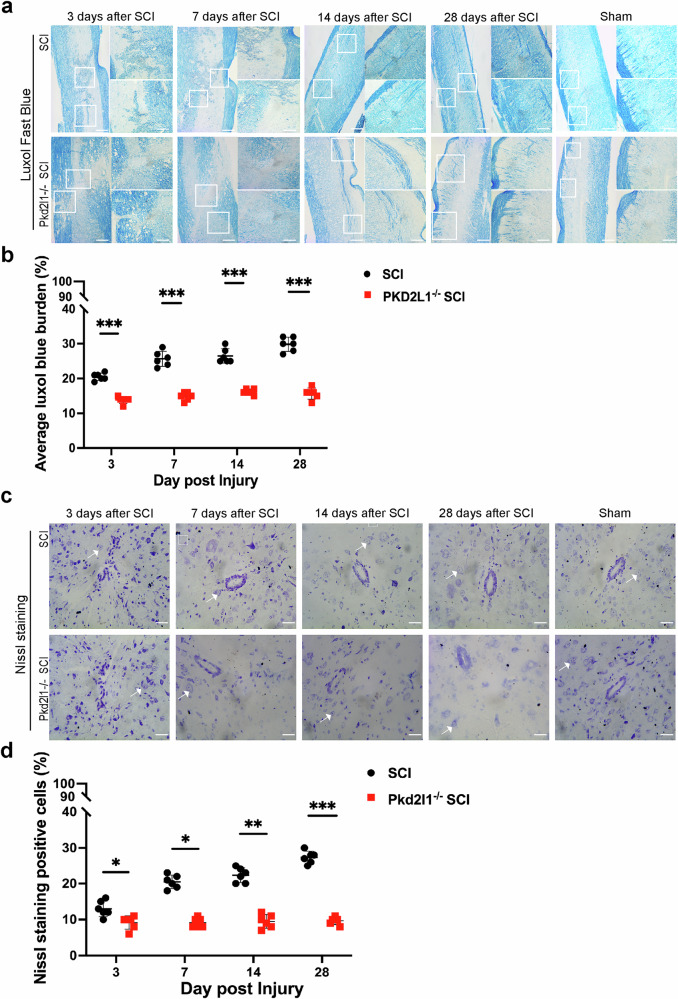
Pkd2l1 deletion retards myelin and neuronal regeneration following SCI. **a**, **b** Luxol Fast Blue staining showed that myelin regeneration was almost depleted in Pkd2l1^−/−^ mice (*n* = 6). **c**, **d** Nissl staining showed that Pkd2l1^−/−^ mice had a notable reduction in Nissl bodies surrounding the central canal following SCI (*n* = 6). *p < 0.05, **p < 0.01, ***p < 0.001. Data are presented as the mean ± SD: scale bars, 100 µm.

Since NSCs are involved in injured spinal cord repair and CSF-cN ablation by Pkd2l1 deletion suppresses cell proliferation, we assumed that neural regeneration and repair at the site of SCI would be impaired in Pkd2l1^−/−^ mice. Myelin loss is a pathological hallmark of SCI that contributes significantly to neuronal functional deficits after SCI. Luxol Fast Blue staining was employed to visualize myelin in neural tissue [33]. The staining results showed that Pkd2l1^−/−^ mice had lower myelin density than WT mice (*p* < 0.001, *n* = 6, Fig. 5a, b), indicating that the deletion of Pkd2l1 suppresses myelin formation.

Nissl bodies are specialized granules in the cytoplasm of nerve cells involved in protein synthesis and proper neuronal function [34]. Nissl staining results of tissue slices in spinal cord showed the formation of Nissl bodies was inhibited in Pkd2l1^−/−^ mice compared to WT mice (*p* < 0.05, *n* = 6, Fig. 5c, d), indicating that the deletion of Pkd2l1 suppresses protein synthesis and proper neuronal function. These results suggest that the knockout of Pkd2l1 impedes the structural changes and proper functioning of neurons surrounding the central canal of the spinal cord after SCI.

**Fig. 6:**
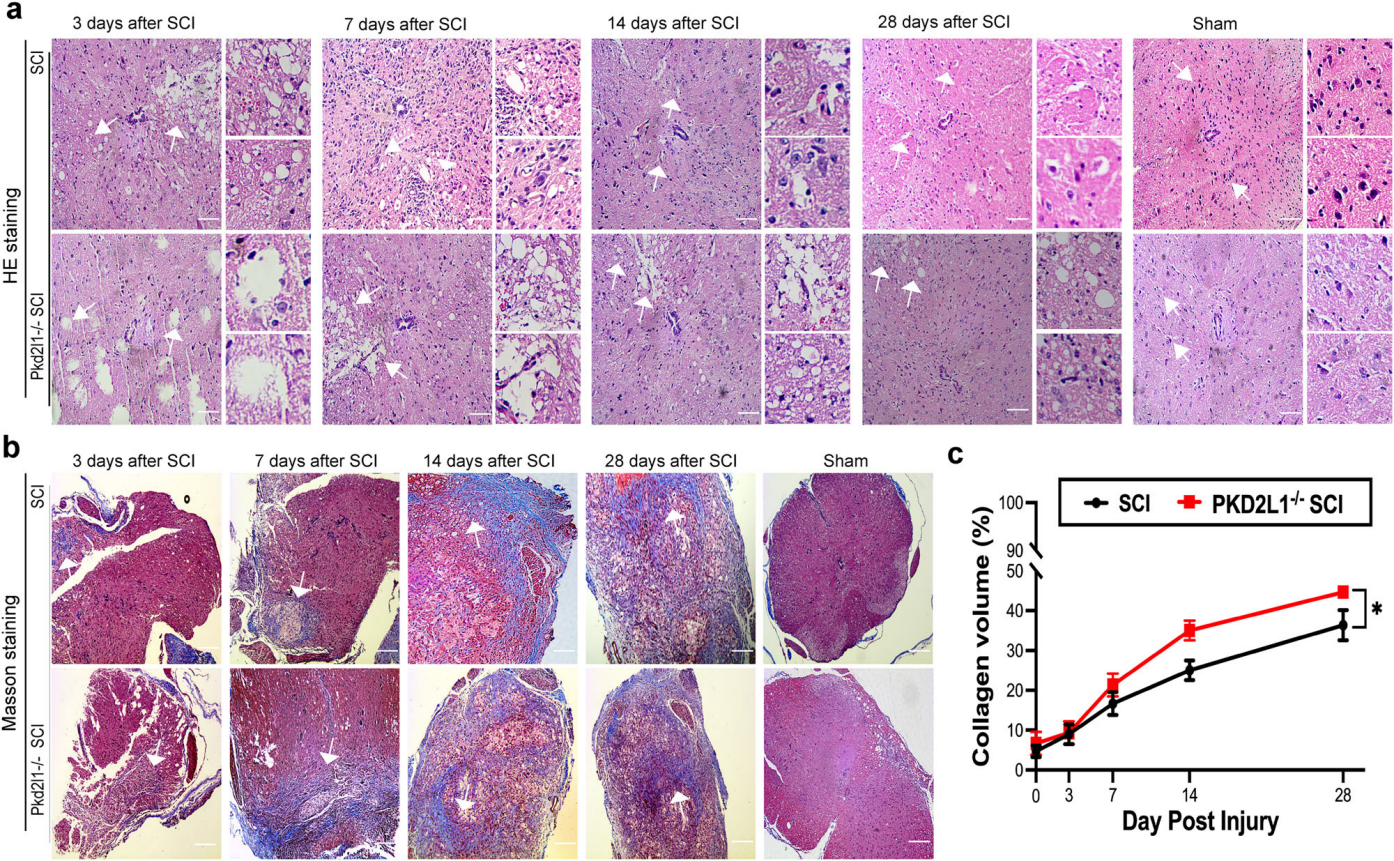
Pkd2l1 deletion promotes pericentral canal vacuolar degeneration and glial scar formation following SCI. **a** H&E staining showed that Pkd2l1^−/−^ mice had more pericentral canal vacuolar degeneration after SCI. **b**, **c** Masson staining conducted on the transverse section of spinal cord tissue at the thoracic 10 (T10) level revealed that Pkd2l1^−/−^ mice exhibited pronounced accumulation of collagen fibers, leading to the formation of glial scars around the central canal following SCI (*n* = 6). *p < 0.05. Data are presented as the mean ± SD. Scale bars, 100 µm.

Vacuolar degeneration in neurons, which is typically seen in neurodegenerative diseases, refers to the abnormal accumulation of vacuoles within the cytoplasm of nerve cells. Hematoxylin and eosin staining of the central canal revealed more vacuoles in Pkd2l1^−/−^ mice than in WT mice after SCI, indicating that Pkd2l1 deletion promotes vacuolar degeneration of neurons surrounding the central canal (Fig. 6a).

The glial scar, formed predominantly by astrocytes, is a significant contributing factor to the failure of spinal cord regeneration after SCI [35]. To elucidate the role of Pkd2l1 in glial scar formation after SCI, we measured the accumulation of collagen fibers by Masso’s trichrome staining. The amount of collagen fibers surrounding the central canal was increased in Pkd2l1^−/−^ mice compared to WT mice after SCI, suggesting that the depletion of Pkd2l1 boosts glial scar formation after SCI (*p* < 0.05, *n* = 6, Fig. 6b, c). Taken together, our results suggest that the depletion of Pkd2l1 promotes vacuolar degeneration and glial scar formation after SCI.

**Fig. 7:**
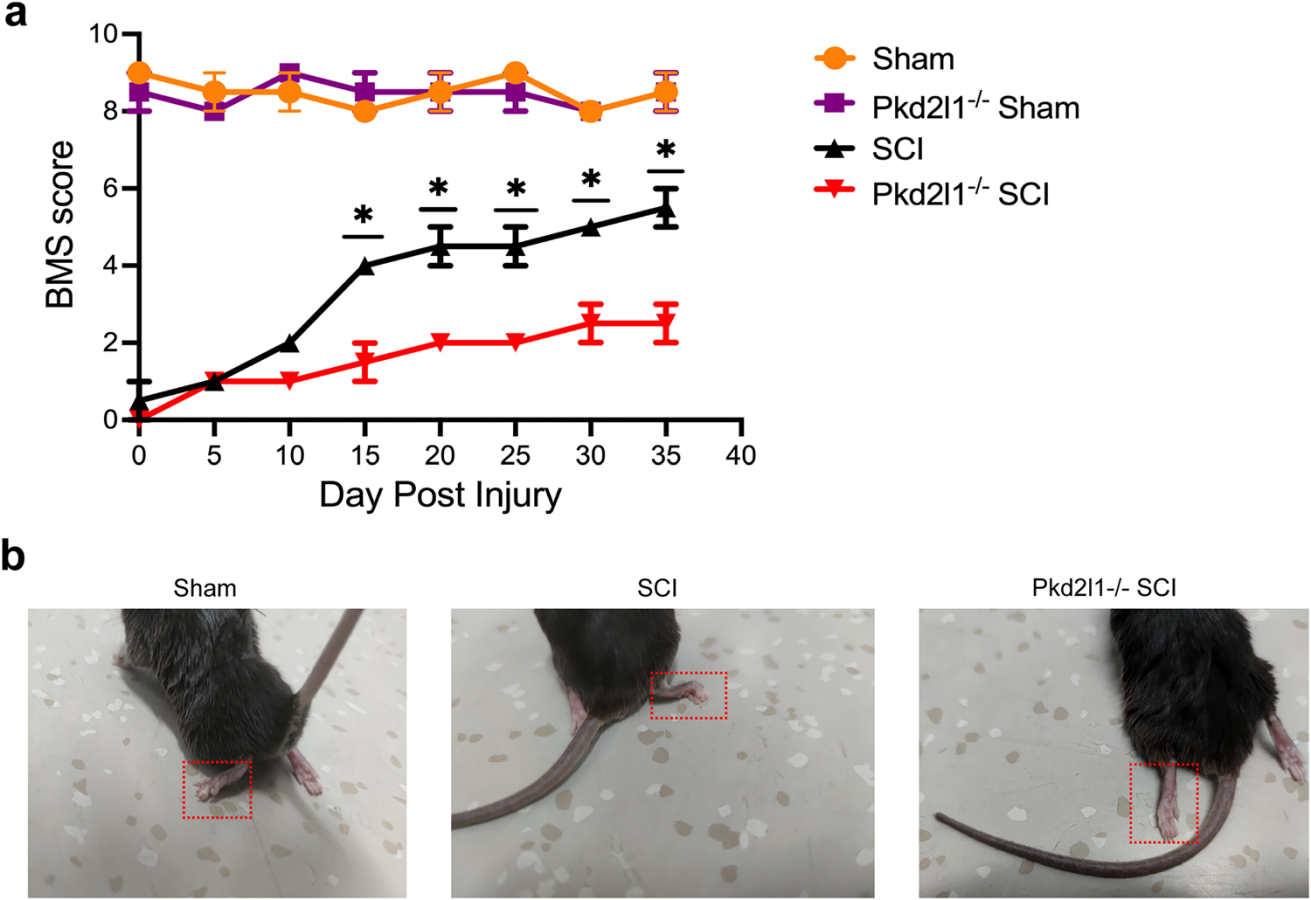
Pkd2l1^−/−^ mice restrained motor function recovery following SCI. **a** Measurement of BMS scores showed that the knockout of Pkd2l1 restricted motor function recovery in mice after SCI. **b** The loss of motor function in Pkd2l1^−/−^ mice was evaluated based on the status of the hind limbs on day 35 following SCI (*n* = 6). *p < 0.05. compared to the Pkd2l1^−/−^ SCI group. Data are presented as the mean ± SD.

Since Pkd2l1 deletion hindered neurogenesis surrounding the central canal by inhibiting myelination and neuron function and promoting vacuolar degeneration and glial scar formation following SCI, we inferred that Pkd2l1 deletion would impair motor function. Hindlimb motor function was quantified using the Basso Mouse Scale (BMS) for locomotion, which evaluates hindlimb movement, forelimb–hindlimb coordination, and trunk stability [36, 37]. Evaluation of the BMS score showed that without SCI, Pkd2l1^−/−^ mice and WT mice showed no significant difference in hindlimb motor function. However, Pkd2l1^−/−^ mice displayed poorer hindlimb motor function than WT mice following SCI (*p* < 0.05, *n* = 6, Fig. 7a, b). These results confirm that Pkd2l1 deletion hinders hindlimb motor function after SCI. Taken together, our results demonstrate that Pkd2l1 depletion inhibits myelin formation and neuron function, promotes vacuolar degeneration and glial scar formation, and hinders hindlimb motor function after SCI.

## Discussion

In this study, we explored the role of the Pkd2l1 gene in the neurogenesis of CSF-cNs and injured spinal cord repair. We found that Pkd2l1 gene knockout almost completely depleted the CSF-cN population, indicating that Pkd2l1 is a key regulator of CSF-cN neurogenesis. Then, we found that the near complete depletion of NSCs occurred concomitant with the observed decline in CSF-cNs, indicating that CSF-cNs are possibly the main source of NSCs surrounding the central canal of the spinal cord. Furthermore, Pkd2l1^−/−^ mice displayed deficient neurogenesis and impaired motor recovery after SCI. Thus, our results suggest that Pkd2l1 is involved in the neurogenesis of CSF-cNs and NSCs surrounding the central canal, indicating that the manipulation of Pkd2l1 expression in CSF-cNs has implications for injured spinal cord repair.

We found that Pkd2l1 plays a significant role in regulating the number of CSF-cNs in the spinal cord. Pkd2l1 has been identified as a marker for CSF-cNs in the spinal cord of vertebrates, a discovery that has facilitated the creation of stable transgenic lines in mice and zebrafish, laying a solid foundation for investigating the physiological properties of CSF-cNs [16]. Studies in Pkd2l1 knockout zebrafish mutants have demonstrated that Pkd2l1 is essential for the mechanosensory function of CSF-cNs [20]. Additionally, research on Pkd2l1 mutant mice has revealed that CSF-cNs contribute to the precise execution of movements required for skilled motor coordination, and Pkd2l1 is indispensable for maintaining the normal functionality of CSF-cNs in mice [38]. Our previous studies confirmed that CSF-cNs have NSC properties in vitro and that SCI or VEGF+bFGF injection triggers CSF-cN division as NSCs in vivo [14, 15]. Therefore, we postulated that Pkd2l1 deletion in mice would suppress the neurogenesis of CSF-cNs after SCI. As expected, Pkd2l1 deletion in transgenic mice resulted in the disappearance of CSF-cNs, as confirmed by expression of the CSF-cN markers GABA, Gata2, and Gata3 and a failure to proliferate after SCI. Nevertheless, Gerstmann’s research showed that Pkd2l1 deletion did not affect the presence or locomotor function of CSF-cNs, but this previous study did not provide evidence confirming the existence of CSF-cNs after Pkd2l1 deletion [38]. In addition, Pkd2l1, as a calcium-selective ion channel, regulates Ca^2+^ influx, and intracellular Ca^2+^ dynamics are closely associated with the survival and activation of NSCs [24, 28, 39–41]. Calcium waves were found to increase the proliferation of radial glial cells, which function as neuronal progenitor cells, in the cortical ventricular zone, and disruption of calcium waves decreases ventricular zone proliferation during embryonic neurogenesis [41]. The transition of NSCs from a quiescent state to a proliferative state was found to rely on Ca^2+^ dynamics, and higher steady-state cytosolic intracellular Ca^2+^ levels can activate NSCs. In addition, EGF triggers NSC division through Ca^2+^ dynamics and higher cytosolic Ca^2+^ levels [24]. These results show that Ca^2+^ signaling is a vital regulator that determines the proliferation of NSCs [41–44]. Although this study highlights the critical role of Pkd2l1 in CSF-cN neurogenesis, the downstream signaling pathways involved in Pkd2l1-mediated regulation of CSF-cN proliferation and differentiation remain unclear. Further investigation into the mechanisms by which Pkd2l1 governs CSF-cN neurogenesis will be essential to advancing strategies for manipulating neurogenesis following SCI.

Our results suggest that CSF-cNs are possibly the primary population of NSCs in the spinal cord. Previous research has shown that CSF-cNs exhibit higher levels of numerous molecular markers of immature neurons, such as Nkx6-1, Sox2, neuronal β-III tubulin and Dcx, indicating that Pkd2l1^+^ cells comprise an immature neuronal population [16, 26, 27]. We previously found that CSF-cNs expressed high levels of the neural progenitor cell markers Nestin, Sox2, β-III tubulin and Dcx in vivo. In vitro, we confirmed that mouse CSF-cNs formed neurospheres, an indicator of NSCs, and had multilineage differentiation potential and the ability to differentiate into neurons, astrocytes, and oligodendrocytes [14]. In vivo assays showed that SCI activated CSF-cNs to generate NSCs and triggered the proliferation of CSF-cNs [15]. These findings demonstrated that CSF-cNs act as NSCs and thus may contribute to the spinal response to injury. Intriguingly, the disappearance of CSF-cNs caused by Pkd2l1 deletion resulted in the loss of NSC activation and proliferation after SCI in the region surrounding the central canal, strongly supporting the idea that CSF-cNs may be the main population of NSCs in this region. Consistent with our findings, Skinnider et al. used mononucleotomics in mice to compare the proportion of neurons in each subpopulation in the damaged and undamaged spinal cords, and found that the only exception was neurons exposed to CSF-cNs, which showed unique resilience to SCI [17]. Consequently, the loss of CSF-cNs retarded myelin and neuron regeneration, boosted pericentral canal vacuolar degeneration and glial scar formation, and restrained motor function recovery following SCI, suggesting that CSF-cNs act mainly as NSCs after SCI. Collectively, these results demonstrate that CSF-cNs are the primary source of NSCs in the spinal cord and hold significant promise for regenerative therapies after SCI.

SCI causes neuron death and leads to permanent functional impairment. Currently, no curative treatments for complete functional recovery are available [45–47]. However, adult NSCs have gradually garnered attention as potential therapeutic targets for SCI. Inducing the regeneration of adult NSCs has emerged as a promising strategy for SCI treatment, offering unique advantages [1, 48, 49]. Nanotechnology-based biomaterials, such as nanoparticles, tissue-engineered nerve guidance conduits, and core–shell structured fibers with acellular nerve matrix as the shell and poly(ε-caprolactone) as the core, have demonstrated remarkable efficacy in promoting rapid and targeted nerve regeneration [50–52]. This study indicates that CSF-cNs may serve as a primary source of NSCs following SCI. Utilizing advanced biomaterials to induce the self-renewal of CSF-cNs and promote neural repair after SCI provides new insights and directions for CSF-cN-based therapeutic strategies. Furthermore, Deletion of the Pkd2l1 gene in transgenic mice eliminated the population of CSF-cNs, which resulted in the deficient proliferation of NSCs surrounding the central canal and retarded neurogenesis and functional recovery after SCI. Pkd2l1, as a pivotal regulator of CSF-cNs proliferation, may activate the self-renewal of CSF-cNs and promote neural repair in vivo. This mechanism could provide an effective regulatory target for utilizing endogenous NSCs in the treatment of SCI.

## Materials and methods

### Animals

Female C57BL/6 mice aged 6–8 weeks were provided by the Experimental Animal Center of Guizhou Medical University. The mice were divided into two cohorts, namely, the Pkd2l1 deletion and Pkd2l1 wild type (WT) strains. Pkd2l1 WT mice were acquired from Beijing Viton Lihua Laboratory Animal Technology Co., Ltd. Pkd2l1 knockout mice were purchased from Cyagen Biosciences (Guangzhou, China). Animals had free access to food and water throughout the study and were group-housed in standard Plexiglas cages on a 12-h light/12-h dark cycle (light cycle = 6:00 a.m.–6:00 p.m.). Animal housing facilities had an ambient temperature of 20–23 °C with 30–70% humidity.

### SCI model

The dorsal column lesion model of SCI was utilized for this study for ease of animal care as previously described [15]. Dorsal column lesions were made at the thoracic spinal cord level 10 (T10). All surgeries were performed under deep anesthesia using a combination of ketamine (25 mg/kg), xylazine (5.8 mg/kg), acepromazine (0.25 mg/kg), and inhaled isoflurane (0.5–1%). Following laminectomy at T10, a tungsten wire knife with an extruded diameter of 1.0–1.5 mm (McHugh Milieux, Downers Grove, IL) was centered above the spinal cord midline, retracted, and inserted to a depth of 0.8 mm below the dorsal spinal cord surface. The arc of the knife was then extruded and raised to transect the spinal cord dorsal columns. A calibrated aneurysm clip with a force of 30 g was utilized to induce a 30-second constriction of the spinal cord at the tenth thoracic vertebra level [53, 54]. The clip was opened using a clamp holder and carefully passed through the T10 spinal cord. Subsequently, the clip was abruptly released, resulting in a sudden and severe injury to the spinal cord. The group that was administered a placebo did not experience any cord compression by the clip. We kept the mice warm until awakening and placed some food in the cage after SCI. We manually emptied mice bladders twice a day and monitored the clinical signs.

### Tissue immunofluorescence

The spinal cord tissue was fixed overnight in 4% paraformaldehyde (PFA) at 4 °C. Next, gradient dehydration was conducted using 30% sucrose, and the tissue was then embedded in OCT. Frozen spinal cord sections (20-µm thick) were cut in a transversal or longitudinal (1-cm long) plane. For imaging, transverse sections from the cervical, thoracic, and lumbar regions, as well as longitudinal sections spanning the cervical to lumbar regions, were used. Subsequently, the specimens were treated with a solution containing 0.25% Triton X-100 for 10 min, followed by blocking with 1% BSA for 1 h. The sections were incubated with primary antibodies against the following proteins at 4 °C: Pkd2l1 (AB9084, 1:700, Millipore, USA), Nestin (sc23927, 1:200, Santa Cruz Biotechnology, Dallas, TX, USA), Sox2 (ab79351, 1:500, abcam, USA), Dcx (ab153668, 1:200, abcam, USA), β-III tubulin (66240-1-Ig, 1:200, Proteintech, USA), GFAP (16825-1-AP, 1:300, Proteintech, USA), Foxj1 (sc-53139, 1:200, Santa Cruz Biotechnology, Dallas, TX, USA), GABA (A2052, 1:200, Millipore, USA), Gata2 (11103-1-AP, 1:300, Proteintech, USA), and Gata3 (66400-1-Ig, 1:300, Proteintech, USA). After being washed three times with PBS, the tissue sections were incubated in the dark for one hour with secondary antibodies (goat anti-rabbit Alexa Fluor 488, 1:500, CST, Danvers, MA, USA; or goat anti-mouse Alexa Fluor 594, 1:500, CST, Danvers, MA, USA). Additionally, the sections were counterstained with DAPI.

For histological analysis, the immunostained sections were examined under a Leica confocal microscope. The number of cells positive for each marker was quantified in at least three random fields per section, with a minimum of three sections analyzed per animal. The percentage of cells expressing each marker was calculated relative to the total number of DAPI-positive cells. Colocalization analysis was performed to determine the expression of multiple markers within the same cells. Signals were detected and images were obtained utilizing a Leica confocal microscope.

### 5‐Ethynyl‐2′‐deoxyuridine staining and imaging

Frozen spinal cord sections (5-µm thick) were cut in a longitudinal (1-cm long) plane. To facilitate the identification of cells undergoing proliferation as a result of SCI or growth factor infusions, 50 mg/kg 5-ethynyl-2-deoxyuridine (EdU) (C00053, RioBio, Guangzhou, China) was administered to the mice through intraperitoneal injection. The subsequent methodology employed in this experiment was similar to that used to process sections for immunofluorescence. After immunofluorescence staining, the sections were stained for EdU using the cell-light EdU Apollo 567 reagent (C10310-1, RioBio, Guangzhou, China) according to the manufacturer’s instructions. The resulting signals were visualized using a Leica confocal microscope.

### Hematoxylin and eosin staining

Hematoxylin and eosin (H&E) staining was used to observe spinal cord tissue repair. The mice were euthanized, and their spinal cords were collected and preserved in 10% phosphate-buffered formalin for 24 h. Spinal cords were embedded in paraffin and serial sections (5-µm thick) were cut in a transversal plane. The sections were then stained with a modified hematoxylin and eosin (H&E) staining reagent (G1120, Solarbio, Beijing, China) and examined using a microscope. Photographs were taken to evaluate the morphological restoration of the spinal cord tissue at the injury site.

### Luxol fast blue staining

Luxol fast blue (LFB) staining was used to observe myelin damage of spinal cord tissue. Spinal cords were embedded in paraffin and serial sections (5-µm thick) were cut in a longitudinal (1-cm long) plane. The tissue sections were subjected to dewaxing and subsequently stained using a Luxol Fast Blue Myelin Stain Kit (G1030, Servicebio, Wuhan, China). After dehydration and coverslipping, the sections were examined under a microscope and photographed to evaluate any changes in myelin composition within the spinal cord and adjacent tissues surrounding the site of injury. The area of Luxol fast blue was quantified by ImageJ analysis software.

### Nissl staining

Spinal cord ischemia is correlated with neuronal degeneration in the ventral horn of the lumbar spinal cord, and the viability of these affected neurons can be evaluated through the utilization of Nissl staining. Spinal cords were embedded in paraffin and serial sections (5-µm thick) were cut in a transversal plane. The spinal cord sections were cleansed using distilled water and then incubated for 1 h at 37 °C in Nissl staining solution (G1430, Solarbio, Beijing, China). Subsequently, the sections were rinsed with double-distilled water and allowed to undergo Nissl differentiation for 5 s to 2 min. Following dehydration and coverslipping, neuronal images taken using an optical microscope were examined to determine the viability of neurons near the site of spinal cord tissue injury and the surrounding area. Three 100× visual fields were randomly selected and photographed, and the neurons containing Nissl bodies in each field were counted.

### Masson staining

Masson’s trichrome stain was used to determine the levels of collagen deposition. Spinal cords were embedded in paraffin and serial sections (5-µm thick) were cut in a transversal plane. In the process of Masson staining, sections were subjected to staining using the Masson Stain Kit (G1340, Solarbio, China). To perform Masson’s trichrome staining, sections were exposed to a mixture of 10% potassium dichromate and 10% trichloroacetic acid, leading to the hematoxylin-induced coloration of the nuclei. Subsequently, the sections underwent differentiation with hydrochloric acid and ethanol, followed by reduction with a mild ammonia solution to generate a blue hue, and finally, they were stained using Masson’s method. The Masson’s trichrome staining depicted was performed on transverse sections of the mouse spinal cord at the thoracic 10 (T10) level. The blue-stained lesion area was quantified utilizing the ImageJ threshold method.

### Functional behavioral evaluation

The Basso Mouse Scale (BMS) was used to quantify hind limb mobility in mice, as previously described [36]. The BMS score ranges from 0 to 9 and evaluates movement quality in terms of multiple aspects, such as frequency, range of motion, coordination, posterior ankle joint movement, contact between the sole and dorsum of the foot, foot positioning, trunk stability, and tail posture. The evaluators, who were provided with professional guidance, conducted evaluations 3 times and promptly recorded the results without prior knowledge of the experimental conditions. Assessments of the mice commenced on the fifth day following SCI and persisted for seven weeks, consistently occurring at the same weekly time intervals.

### Statistical analysis

Sample size for each animal experiment was predetermined to ensure adequate statistical power for drawing conclusion. Statistical analysis, graphical representation, and data visualization were performed using ImageJ PRO and GraphPad Prism 8.4.0. GraphPad Prism 8 (GraphPad Software, Inc.; La Jolla, CA) was used for statistical analysis. All data are presented as mean ± SEM, except for the BMS scores, which are presented as median ± interquartile range (IQR). For the analysis of BMS scores, the Kruskal-Wallis test was used, followed by Dunn’s post hoc test for comparing BMS scores among multiple groups; the Mann-Whitney U test was used for comparing BMS scores between two groups.

All other data were compared using unpaired t-tests or one-way analysis of variance (ANOVA), with Tukey’s post hoc test applied for group comparisons. All statistical tests were two-sided, and *p < 0.05, **p < 0.01, and*** p < 0.001 were considered statistically significant.

## Data Availability

The raw data supporting the conclusions of this article will be made available by the authors, without undue reservation.
